# Lymphocyte loss and plasmacytosis are associated with IL-6- and TNF-producing cells in the spleens of fatal COVID-19 cases

**DOI:** 10.3389/fcimb.2025.1645378

**Published:** 2025-10-23

**Authors:** Bianca Ramos Mesquita, Lilian Verena da Silva Carvalho, Leonardo Cardoso Gomes Baqueiro, Reginaldo Brito, Luma Bahia Figueiredo Pinto, Erina Masayo Alves Hassegawa, Jonathan Luís Magalhães Fontes, Cláudio Pereira Figueira, Eraldo Salustiano de Moura, Maria Brandão Tavares, Carla Pagliari, Geraldo G. S. Oliveira, Washington L. C. dos-Santos

**Affiliations:** ^1^ Laboratório de Patologia Estrutural e Molecular, Instituto Gonçalo Moniz, Fundação Oswaldo Cruz, Salvador, Brazil; ^2^ Universidade Federal da Bahia, Faculdade de Medicina da Bahia, Salvador, Brazil; ^3^ Instituto Couto Maia, Bahia State Health Secretary, Salvador, Brazil; ^4^ Hospital do Subúrbio, Bahia State Health Secretary, Salvador, Brazil; ^5^ Departamento de Patologia, Universidade de São Paulo, Faculdade de Medicina, São Paulo, Brazil

**Keywords:** spleen disorganization, TNF, IL-6, lymphocyte loss, COVID-19

## Abstract

**Background:**

The spleen undergoes changes during acute and chronic infections, which may contribute to immune dysregulation and disease aggravation. In fatal cases of COVID-19, pronounced splenic changes are noted. However, the role played by these alterations in patient mortality remains poorly understood. Objectives: We aim to characterize structural alterations and changes in splenic cell populations in fatal COVID-19 cases, as a potential substrate for immune dysfunction associated with bacterial coinfection and mortality in severe infectious diseases.

**Methods:**

In this study, we characterized the histological and cellular changes observed in the spleens of nine patients who died from COVID-19. Spleens from five healthy individuals were used as a reference. Histopathological analysis and immunolabeling techniques were employed to evaluate tissue architecture, cell composition, cytokine production, and cell death.

**Results:**

COVID-19-associated changes included atrophy of the white pulp (WP), reduced cellular density in the red pulp (RP), and reticular fiber fragmentation. Leukocyte phenotyping revealed substantial lymphocyte depletion across all splenic compartments, accompanied by plasma cell accumulation. These alterations correlated with increased numbers of IL-6- and TNF-producing cells. Additionally, a high density of TUNEL-positive cells indicated widespread cell death in the spleens of COVID-19 patients.

**Conclusion:**

These findings suggest that the spleen contributes to the inflammatory response in *SARS-CoV-2* infection, acting both as a source of inflammatory cytokines as well as a site of leukocyte, particularly lymphocyte, death both in association with the exacerbated release of IL-6 and TNF.

## 1 Introduction

The spleen is a secondary lymphoid organ responsible for surveillance against pathogens circulating in the blood (Bohnsack and Brown, 1986). Its microscopic structure consists of two compartments: white pulp (WP) and red pulp (RP), with a marginal zone (MZ) between the WP and RP. The splenic compartments undergo substantial changes during infections (Mebius and Kraal, 2005). The WP, responsible for initiating the immune response, usually presents lymphoid follicle (LF) hyperplasia and germinal center (GC) formation. Among other changes characteristic of different diseases, the memory cell pool may increase in the MZ, while the plasma cell population can rise in the RP (Hermida et al., 2018; Lewis et al., 2019).

In long-lasting infections, such as visceral leishmaniasis (VL), the spleen presents sequential changes that result in a large replacement of cell populations, followed by hyperplasia and/or atrophy, as well as the disruption of splenic compartments (Santana et al., 2008; Silva et al., 2012; Silva-O’Hare et al., 2016; Melo et al., 2021). In acute severe infections, such as bacterial sepsis, changes in the spleen are mostly associated with the elimination of cell populations by different mechanisms of cell death (Hotchkiss et al., 2001; Toti et al., 2004; Ihlow et al., 2021).

While some knowledge has been accumulated on spleen changes in chronic diseases and how this organ may be involved in the maintenance of chronic infections (Hermida et al., 2018; Lenti et al., 2022), little is known about splenic alterations and associated consequences in the context of acute severe infections. An interesting study by Huston et al. (2008) showed that inhibiting cell death in the spleen prevented death in a murine model of experimental sepsis (Huston et al., 2008). This observation was further supported by treatment with cell death inhibitors, such as IL-7 and anti-PD-L1 (Cao et al., 2019). Thus, it follows that understanding how acute severe infections provoke changes in the spleen may aid in designing more effective strategies to better manage patients.

From 2019 to 2022, a disproportionate number of individuals died from COVID-19. The infection, caused by the *SARS-CoV-2*, evolved with severe acute respiratory syndrome, accompanied by coagulopathy and high immune system activation (Iba et al., 2020; Zanza et al., 2022). In the beginning of this period, approximately 20% of patients with severe COVID-19 died, with a mean time between disease onset and death lasting 17 days (Byrne et al., 2020; Dorjee et al., 2020; Linton et al., 2020). Some autopsy studies indicated that the spleen was severely affected by COVID-19 (Duarte-Neto et al., 2020; Liu et al., 2020). However, no systematic studies have reported on the spleen changes evidenced by *SARS-CoV-2* infection, nor speculated about possible implications regarding the course of disease.

Using a minimally invasive autopsy technique, we had the opportunity to collect and study spleen samples from nine patients who died of COVID-19. This study reports profound disorganization of spleen compartments due to severe COVID-19, associated with the decreased density of cell populations. We further endeavored to examine the potential pathways involved in this observed disorganization, including cytokine expression and changes in the extracellular matrix that may have disrupted leukocyte distribution in the organ.

## 2 Materials and methods

### 2.1 Ethical statement

This study is in conformance with the ethical guidelines approved by the National Research Ethics Committee (CONEP), registered under protocol no 4.526.485. The relatives who were legally responsible for the patients were contacted following their death and signed a Free Informed Consent Form.

### 2.2 Patients and spleen samples

Fourteen patients who died from COVID-19 at the Couto Maia Institute (ICOM, Salvador, Bahia-Brazil) between 2021 and 2022 were submitted to ultrasound-guided minimally invasive autopsies. As spleen sample collection was unsuccessful in five of these patients, the present findings are based on samples obtained from the remaining nine patients.

All patients had tested positive for *SARS-CoV-2* by RT-PCR conducted on nasal swab samples prior to death. Autopsies were performed between five and eight hours after death. Specimens were preserved for study by optical microscopy. Samples from five spleens surgically removed from patients who underwent therapeutic splenectomies due to trauma at another institution in the same city, Hospital do Subúrbio, were used as controls (CT).

### 2.3 Histological processing

All collected spleen samples were fixed in paraformaldehyde or formalin, then dehydrated and embedded in paraffin. Following preparation for histological processing, all slides were scanned using a Zeiss-Axio Imager.Z2 (Zeiss, Germany) and viewed using VSviewer software. Images selected for publication were digitally adjusted in Photoshop, when necessary, using levels, contrast, sharpness, and/or color balance tools to enhance clarity. No specific areas of the images were highlighted or obscured.

#### 2.3.1 Histochemistry

Four micrometer-thick spleen sections were stained with hematoxylin and eosin (H&E), periodic acid silver methenamine stain (PAMS) and Prussian blue stain (Perls).

#### 2.3.2 Immunohistochemistry

Spleen sections were mounted onto silanized histological slides. The sections were dewaxed through consecutive immersion in xylene, rehydrated using decreasing concentrations of alcohol (100% → 90% → 70%), and then immersed in distilled water. The hydrated slides were subjected to heat-induced antigen retrieval by immersion in Tris-EDTA buffer (pH 9.0) followed by heating in an electric pressure cooker at 115°C under 70 kilopascals (kPa) for 20 minutes. Next, the slides were allowed to cool to room temperature for 20 minutes while still immersed in the antigen retrieval solution. To block endogenous peroxidase, the slides were treated twice with 3% hydrogen peroxide for 10 minutes, followed by 20 minutes of incubation in 2.5% horse serum (HRP Polymer Detection Kit, Vector Laboratories, California, United States) to block nonspecific binding. Slides were washed with distilled water or 1x phosphate-buffered saline (PBS) between each step of the staining process. Samples were incubated for 16–18 hours at 4°C in a humid chamber with primary antibodies against CD3 (T cells; Abcam- ab16669, 1/200), CD20 (B cells; Abcam- ab64088, 1/200), CD68 (Macrophages; Dako- M0814 1/4000), plasma cells (Dako- M7077, 1/200), IL-6 (ProteinTech- 21865-1-AP, 1/400), TNF (Abcam-ab6671, 1/100), IFN-γ (Byorbit- orb10877, 1/50), IL-10 (Abcam- ab217941, 1/100) and IL-17 (R&D Systems- AF-317-NA, 1/20). After washing with 1x PBS, the slides were incubated with HRP Polymer Detection reagent (Vector Laboratories, United States). As a negative control, some sections were similarly incubated with immunoglobulins of the same isotype and species as the primary antibody. Reaction products were visualized using 3,3-diaminobenzidine solution, while nuclei were counterstained with Harris hematoxylin (Sigma, United States), and slides were finally mounted on Entellan^®^ quick mounting medium (Merck KGA, Germany).

#### 2.3.3 TUNEL staining

Following the previously described deparaffinization and hydration protocol, slides were subjected to antigen retrieval using Proteinase K Antigen Retrieval Solution (ab64220). Subsequently, the sections were incubated with TUNEL reaction mixture provided by the ROCHE *In Situ* Cell Death Detection Kit POD (code: 11684817910) as per manufacturer instructions. The slides were then observed and photographed under an inverted fluorescence microscope (Leica DMi8) using an excitation wavelength of 450–500 nm and a detection range of 515–565 nm (green fluorescence).

### 2.4 Histological analysis

#### 2.4.1 Qualitative analysis

Two trained pathologists blindly conducted the histological qualitative analysis. The classification of white pulp organization was performed according to criteria specified by (Hermida et al., 2018). Spleen sections were stratified into three groups: spleen type 1 or organized, spleen type 2 or mildly disorganized, and spleen type 3 or moderately to extensively disorganized. Plasma cells were identified according to the following criteria: elliptical shape, eccentric nucleus with the characteristic “cartwheel” chromatin pattern, and a prominent perinuclear halo. Plasma cells exhibiting characteristic cytoplasmic inclusions with a grape-like appearance were classified as Mott cells (Allen and Sharma, 2022). Iron deposition was evaluated semi-quantitatively in Perls’-stained. The analysis focused on the red pulp (RP), and staining intensity was scored based on the amount and distribution of blue granules as: Mild (sparse or translucent granules occupying <50% of the RP); Moderate (translucent granules in >50% of the RP or dense granules in <50% of the area; Intense: dense granules occupying >50% of the RP. PAMS-stained reticular fibers were classified as normal or abnormal based on size, thickness and integrity.

#### 2.4.2 Morphometry

##### 2.4.2.1 Spleen compartment size

The relative size of spleen compartments was estimated in HE-stained sections using ImageJ software (National Institutes of Health, USA). The ratio of WP versus the total area of the splenic parenchyma was estimated and results were expressed as percentages.

##### 2.4.2.2 Cell populations in spleen compartments

Selected areas of whole scanned images of spleen sections stained with different antibodies were used for morphometric analysis. Selection of RP regions was performed as follows: the area with the highest number of labeled cells was selected for analysis. Subsequently, four additional adjacent non-overlapping areas (above, below, right, and left) were examined. If any of the adjacent areas included WP regions, these were replaced by the nearest non-overlapping area. RP cell counts were performed manually using ImageJ software.

The five largest WP areas presenting central arterioles clearly visible in cross-sections were selected for morphometric analysis. The outer region of the entire WP (LF, PALS), including the MZ, was outlined and measured. Cell counts were manually performed within the delineated area for cytokines, CD68 (Macrophages) and plasma cells. CD3+ T cell counts were carried out in the PALS, while CD20+ B cell counts were performed in the LF and MZ together, where these cell types are typically concentrated.

In both compartments, manual counting provided an absolute number. Cell counts per square millimeter were used to represent cell population density, calculated as [(number of cells/area measured) * 1,000,000]. The average values obtained from five estimates performed in the spleen of each patient were used for statistical analysis.

##### 2.4.2.3 Histochemical and fluorescence staining analyses

Five non-overlapping areas of the RP of spleen sections stained with PAMS were used to analyze perisinusoidal reticular fibers. In each section, the ten largest sinusoid-surrounding fibers were longitudinally measured. The average of these measurements obtained for each patient was used for comparisons between groups.

For TUNEL analysis, images were captured using a 20x objective, with TUNEL-positive cell counts performed in the RP using the particle analysis tool in ImageJ. Counting was supervised, considering particles with a minimum size of 4 µm² and positive fluorescence determined by the Otsu threshold method on a defined scale. Threshold adjustments were made to account for fluorescence variance between experimental samples.

### 2.5 Expression and results analysis

Numerical data are shown as tables and graphs representing absolute values, means, medians or proportions as specified. Statistical significance of differences between groups was tested using Student’s T test or Mann-Whitney for normal or skewed distributions, respectively. For comparisons involving proportions, the Chi-square test with Yates’ correction or Fisher’s exact probability testing were used. For non-parametric correlation analyses, Spearman correlation was applied. The level of significance was established at p<0.05.

## 3 Results

### 3.1 General population characteristics

To study spleen alterations associated with death in COVID-19 cases, spleen samples were analyzed from nine patients who died from COVID-19 and five samples from control (CT) patients. The main characteristics of the COVID-19 patients are shown in **Table 1**. Six patients were male and three were female, with a median age of 65 years. Median illness duration was 23 days, with 12 days of hospital stay. The most common presentations were dyspnea, fever, cough and vomiting. Only one patient had no comorbidities. The laboratory data shown herein, collected within two days of patient death, show lymphopenia with increased neutrophil/lymphocyte ratios, low hemoglobin and hematocrit levels, as well as increased concentrations of blood markers of kidney and liver injury. Demographic data for the CT patients are presented in **Supplementary Table 1**.

**Table 1:** Clinical and laboratory characteristics of COVID-19 patients.

Parameter	Result	Reference value
Clinical parameters:		
Sex	9 (100%)	–
Male (M)	6 (67%)	–
Female (F)	3 (33%)	–
Age (years)	65 [55-71]	–
Time of illness	23 [14.5-25.5]	–
Time of hospitalization	12 [5.5-18]	–
Comorbidities:		–
Diabetes mellitus 2 (%)	5 (56%)	–
Hypertension (%)	7 (78%)	–
Obesity (%)	3 (34%)	–
Laboratory parameters:		
Hemoglobin (g/dL)	10.5 [8.8-14.3]	11 - 14.5
Hematocrit (%)	30,5 [24.9-30.5]	35 - 46
Leucocytes (/mm^3^)	21630 [16005-31320]	4000 - 10000
Lymphocytes (%)	5 [2.8-5.8]	18 - 48
Segmented cells (%)	89 [84-90]	40 - 70
NLR	17.9 [14.8-37.1]	1-3
Monocytes (%)	3 [2.3-4.8]	3-10
Platelets (/mm³) x10³	305 [201-466]	150–450
Creatinine (mg/dL)	2.4 [1.4-3.6]	M: 0.7 - 1.3 F: 0.6 - 1.1
Urea (mg/dL)	139 [115-161]	15 - 45
AST (U/L)	41 [25-67,5]	5-40
ALT (U/L)	63 [32,5-117,5]	7-56

Data expressed as medians with interquartile range. NLR, neutrophil/lymphocyte ratio; AST, Aspartate aminotransferase; ALT, alanine aminotransferase.

### 3.2 Qualitative spleen changes

**Figure 1:**
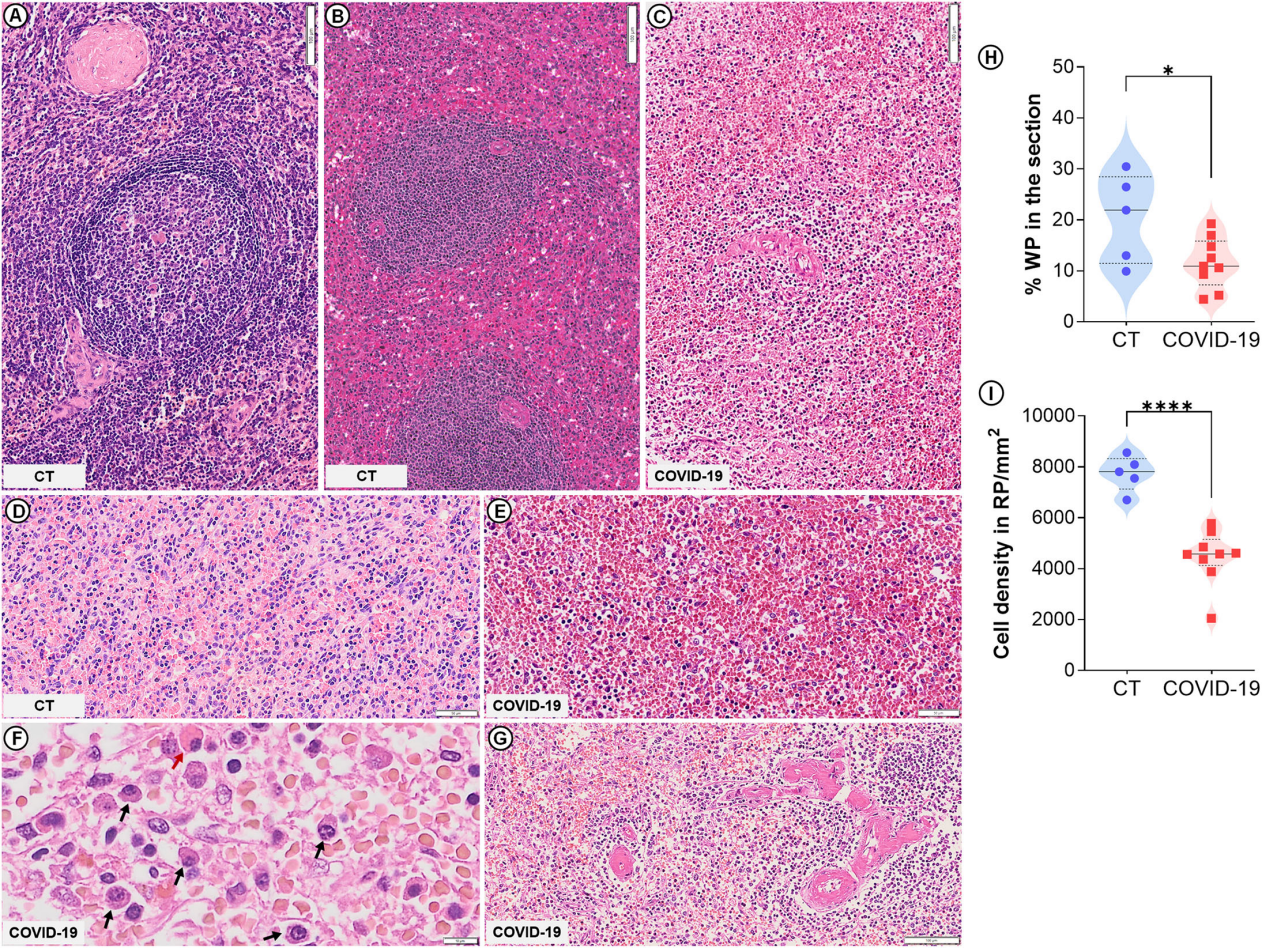
Spleen histological changes in severe COVID-19. Control spleen exhibiting type 1 **(A)** and type 2 **(B)** organization. **(C)** WP disruption (type 3 spleen) evidenced in COVID-19 patients. **(D)** Normal RP density in control patients. **(E)** Low cell density in the RP of COVID-19 patients. **(F)** Plasma cell accumulation (black arrows) and Mott cells (red arrow) in COVID-19 patients. **(G)** Hyaline arteriolosclerosis in COVID-19 patients. **(H)** WP proportion relative to the whole spleen section. **(I)** Total cell density in the RP. Scale bar= 50 and 100 µm.

The spleens of all nine patients who died from COVID-19 were classified as type 3 (moderately to extensively disorganized) (p=0.0009; **Table 2**; **Figure 1C**). In five of these cases, lymphoid follicles were barely visible. The WP was atrophic (p=0.04; **Figures 1C, H**) and low cell density was observed in both RP and WP (p<0.0001; **Figures 1C, E, I**). Three patients presented RP plasmacytosis with some Mott cells (**Figure 1F**), while seven patients presented hyaline arteriolosclerosis (**Figure 1G**).

**Table 2:** Qualitative histological analysis of spleen in patients with COVID-19 and controls.

	COVID-19 (n=9)	CT (n=5)	*P* (<0.05)
H&E			
Type 1 spleen	0/9	3/5	0.0009^a^
Type 2 spleen	0/9	2/5	
Type 3 spleen	9/9	0/5	
PAMS			
Continuous ring fibers	1/9	5/5	0.003^b^
Fragmented ring fibers	8/9	0/5	
Perls			
Mild	3	6	0.08^a^
Moderate	2	0	
Intense	0	3	

a Chi-squared test;

b Fischer’s exact test.

**Figure 2:**
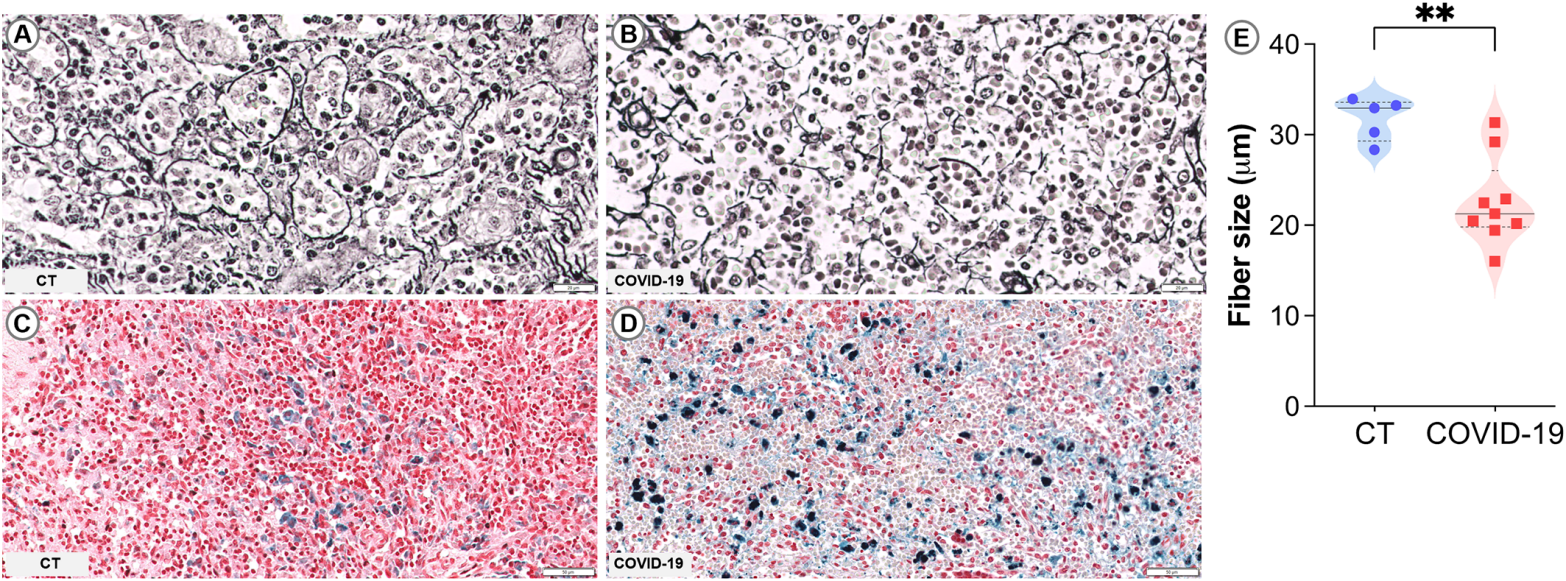
Sinusoidal ring fibers and iron accumulation in COVID-19. **(A, B)** PAMS staining: **(A)** Control spleen showing organized fiber rings. **(B)** COVID-19 spleen with fragmented fiber rings. **(C, D)** Perls staining: **(C)** Control spleen showing no iron accumulation. **(D)** COVID-19 spleen with marked iron accumulation. **(E)** Measurement of the average size of reticular fibers. Scale bar= 20 and 50 µm.

In the spleen, reticular fibers form a framework for cells to attach and move and also help stabilize sinusoids in the RP (Satoh et al., 1997). The reticular fibroblastic cells within the RP continuously produce reticular fibers, primarily composed of type IV collagen and laminin. These fibers are organized into a structure known as “ring fibers” that encircle the sinusoids and are identifiable through silver impregnation due to their argentophilic nature (Apaja‐Sarkkinen et al., 1986). In the cases of COVID-19 observed herein, ring fibers were extensively fragmented and exhibited reduced thickness (p= 0.003; **Table 2**; **Figure 2B**) and smaller average size (p= 0.004; **Figure 2E**) compared to those of CT patients (**Figure 2A**). Intense iron deposits were observed in three COVID-19 patients (**Table 2**; **Figure 2D**) and none of the CT group (**Table 2**; **Figure 2C**).

Among the CT, three had a type 1 spleen (**Figure 1A**) while two had type 2 (**Figure 1B**); no CTs presented any of the spleen changes observed in the COVID-19 patients (**Table 2**). The RP cell density of the CT is shown in **Figure 1D**.

### 3.3 Distribution and density of the main leukocyte populations in spleen compartments

**Figure 3:**
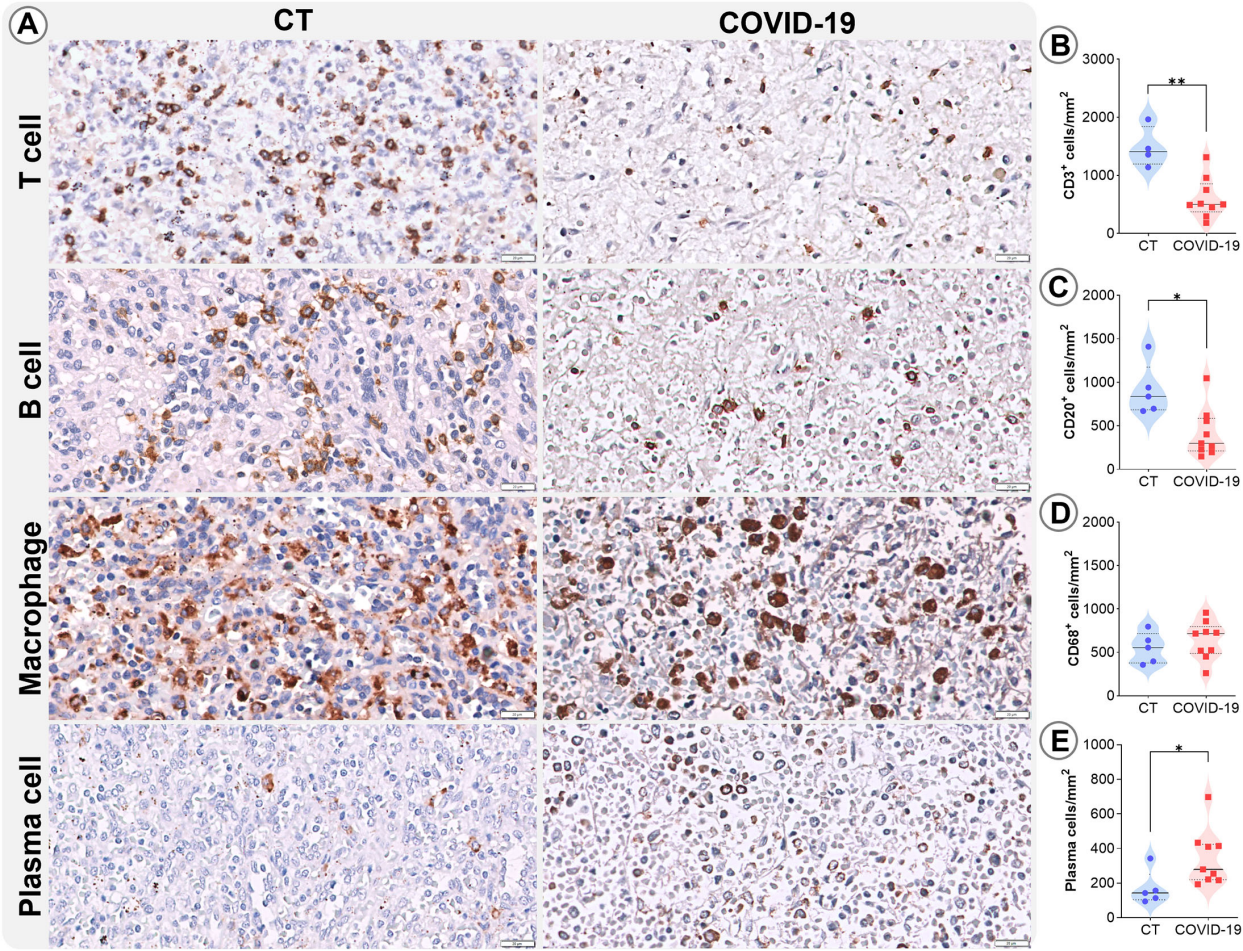
Leukocyte density in the RP. **(A)** Representative micrographs of RP sections stained for T lymphocytes (CD3), B lymphocytes (CD20), macrophages (CD68), and plasma cells in the splenic tissues of control (column 1) and COVID19 patients (column 2). Cell density of **(B)** CD3, **(C)** CD20, **(D)** CD68, and **(E)** plasma cells in the RP of control patients (blue circles) and COVID-19 patients (red squares). *p ≤ 0.05; **p ≤ 0.01. Scale bar= 20 µm.

Patients with COVID-19 exhibited marked reduction in leukocyte populations. Immunohistochemical phenotyping was performed to characterize the main splenocyte subpopulations and identify which cell types were most affected. In the RP, a significant reduction in T cells (p=0.002; **Figures 3A, B**) and B cells (p=0.01; **Figures 3A, C**) were observed, along with increased plasma cells (p= 0.04; **Figures 3A, E**). No significant differences were observed in macrophage populations (**Figures 3A, D**).

**Figure 4:**
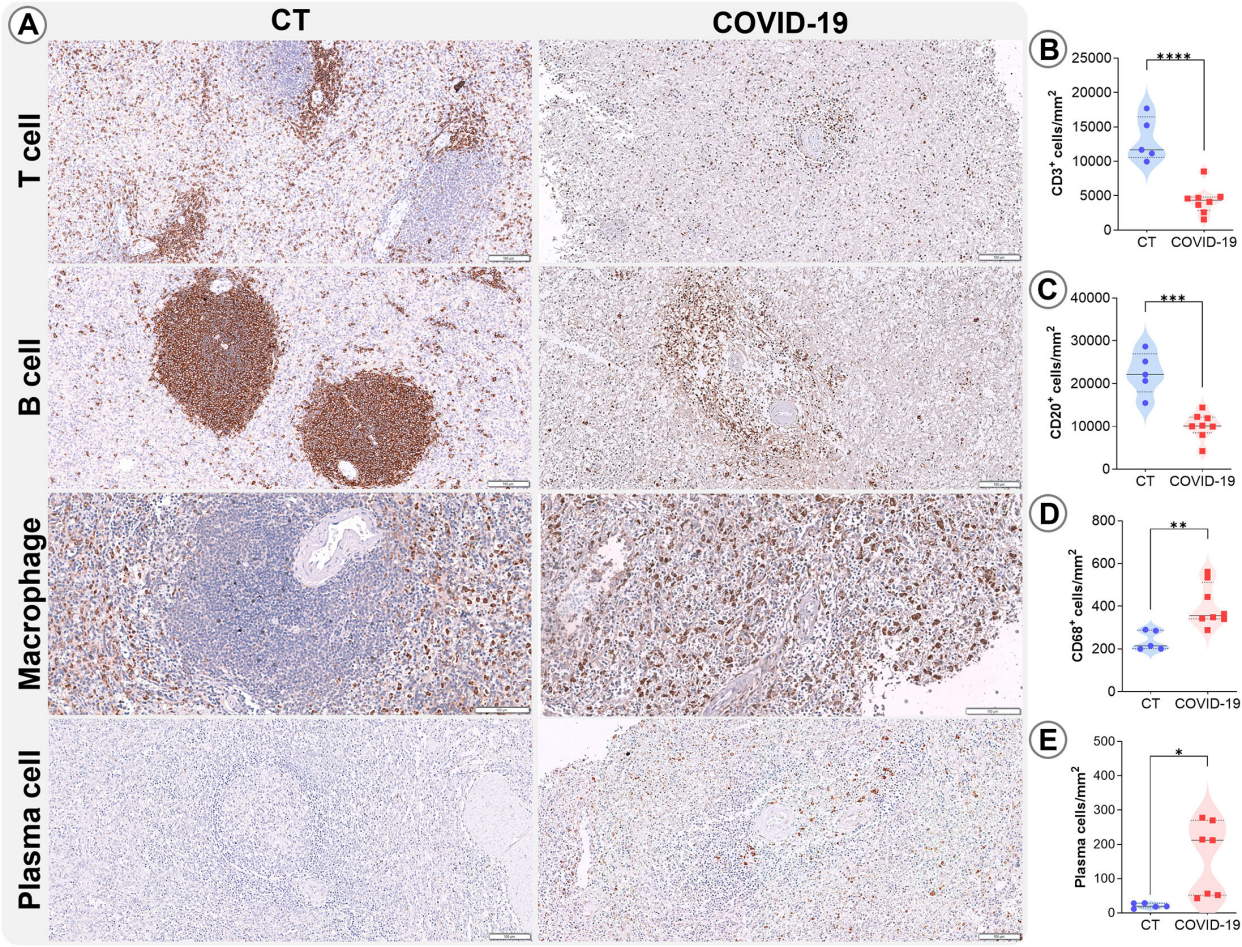
Leukocyte density in the WP. **(A)** Representative micrographs of WP sections stained for T lymphocytes (CD3), B lymphocytes (CD20), macrophages (CD68), and plasma cells in the splenic tissues of control (column 1) and COVID19 patients (column 2). Cell density of **(B)** CD3, **(C)** CD20, **(D)** CD68, and **(E)** plasma cells in the WP of control patients (blue circles) and COVID-19 patients (red squares). *p ≤0.05; **p ≤0.01; ***p ≤0.001; ****p ≤0.0001. Scale bar= 100 µm.

In the WP of COVID-19 patients, low T cell (p<0.0001; **Figures 4A, B**) and B cell (p= 0.0002; **Figures 4A, C**) densities were also observed, as well as increased levels of plasma cells (p= 0.02; **Figures 4A, D**). Additionally, macrophage density was elevated (p=0.003; **Figures 4A, E**) in these patients.

### 3.4 Cytokine-positive cells and cell death in the splenic compartments

**Figure 5:**
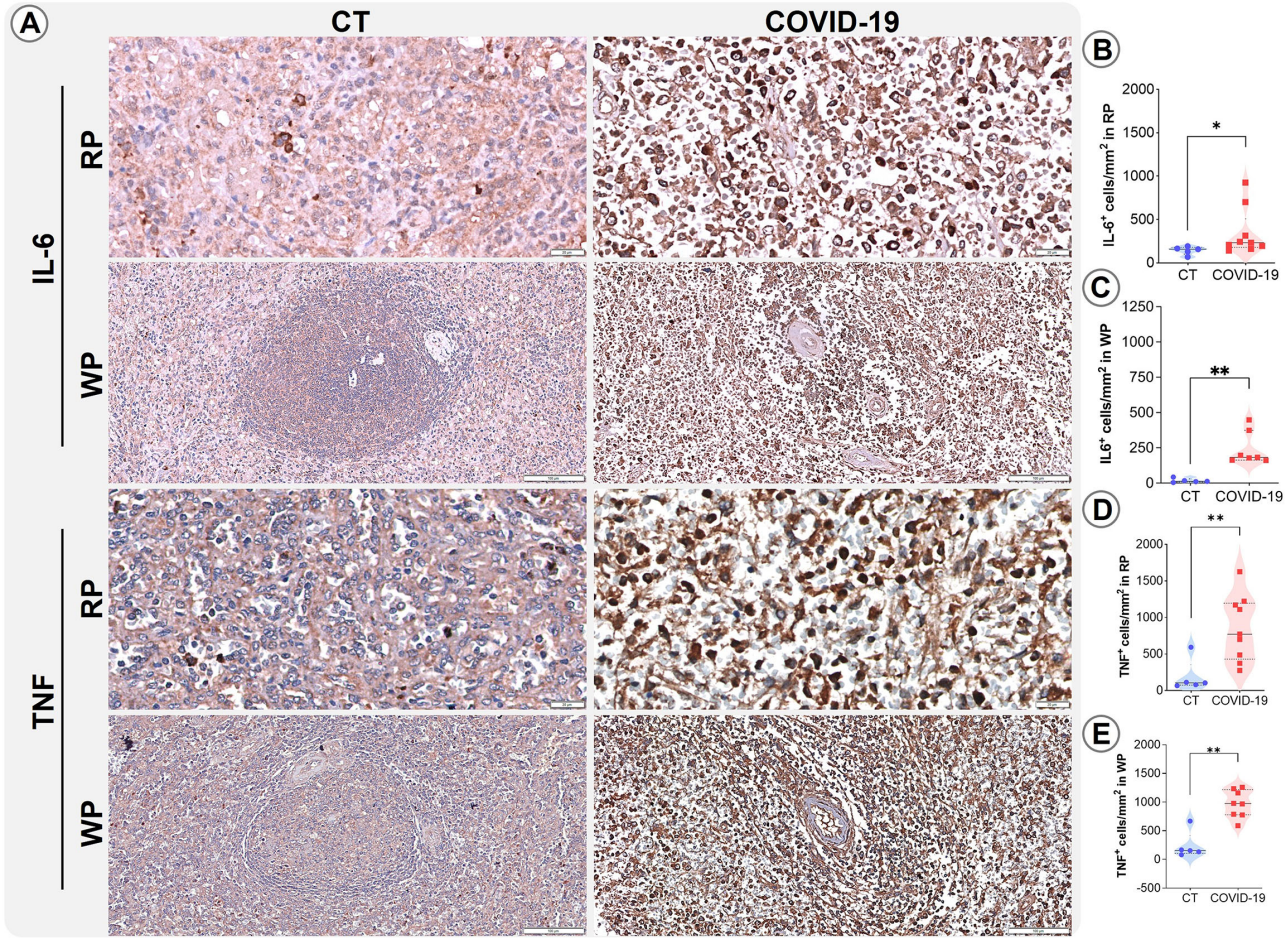
Distribution and density of cells producing IL-6 and TNF. **(A)** Representative micrographs of RP and WP sections stained for IL-6 and TNF in spleens from control (column 1) and COVID-19 patients (column 2). **(B)** IL-6+ cells in the RP, **(C)** IL-6+ cells in the WP, **(D)** TNF+ cells in the RP, and **(E)** TNF+ cells in the WP of COVID-19 patients (red squares) and control patients (blue circles). *p ≤ 0.05; **p ≤ 0.01. Scale bar= 20 and 100 µm.

Severe cases of COVID-19 are known to be associated with the phenomenon referred to as a cytokine storm (Montazersaheb et al., 2022; Zanza et al., 2022). To further investigate the spleen’s involvement in cytokine secretion and consequent impacts on the splenic microenvironments, immunohistochemistry was performed for IL-6, TNF, IFN-γ, IL-10, and IL-17. The pro-inflammatory cytokines IL-6 and TNF were found to be increased in both analyzed compartments (p ≤ 0.05; **Figures 5A–E**) in patients who died from COVID-19. No differences were identified in the density of cells expressing cytokines IL-17, IL-10 or IFN-γ in the RP and WP of the CT and COVID-19 patients analyzed (**Supplementary Figure 1**).

**Figure 6:**
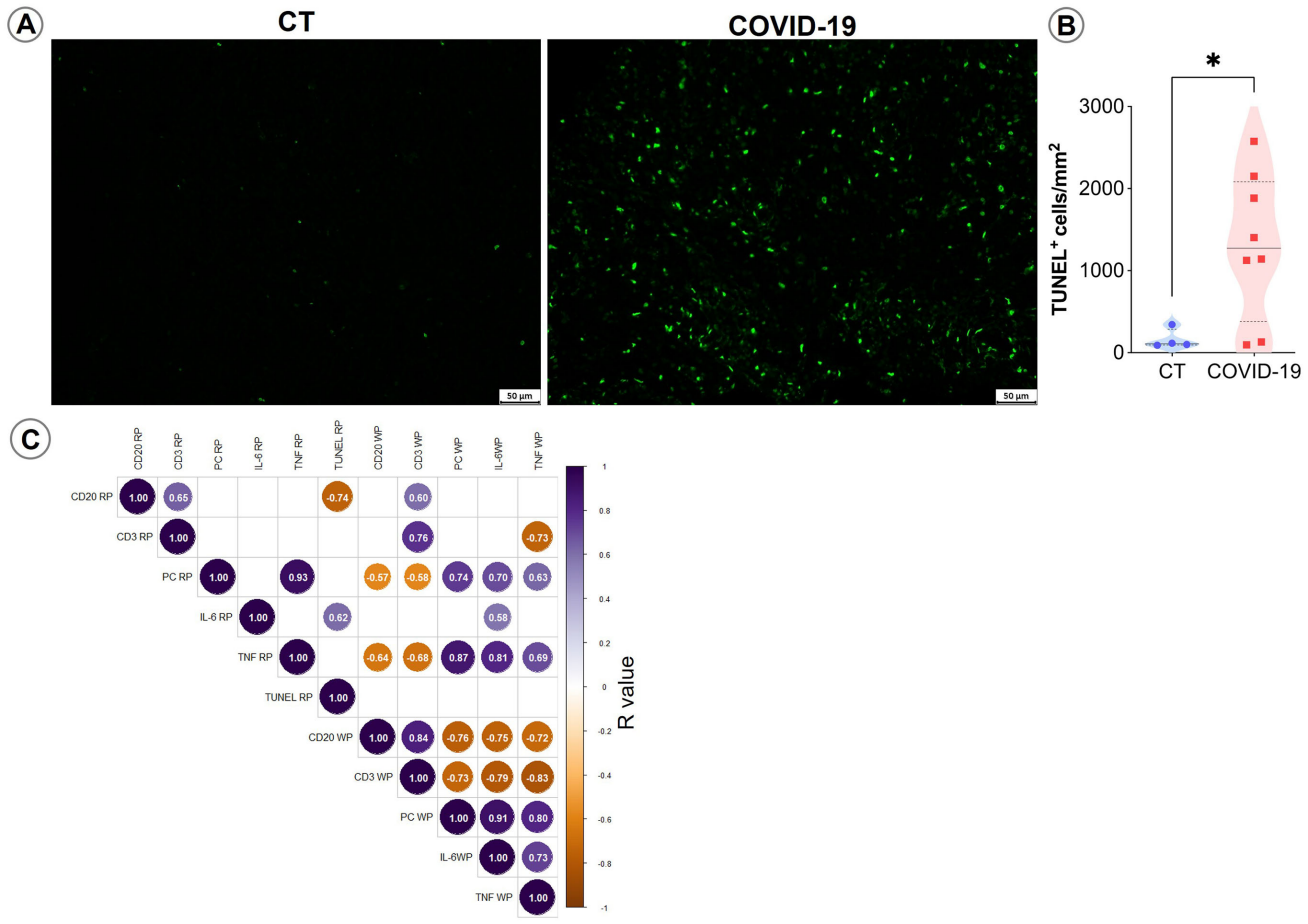
TUNEL staining in spleen sections. **(A)** Representative micrographs of spleen sections stained for TUNEL in control and COVID patients. **(B)** Density of TUNEL-positive cells in the spleens of control (blue circles) and COVID-19 patients (red squares). **(C)** Spearman correlation matrix showing associations between immunohistochemistry counts and TUNEL-positive cells. Only correlations with p ≤ 0.05 are shown.

Increases in IL-6 and TNF cytokine release are usually correlated with cell death. To evaluate whether cell death induction contributed to the observed reductions in cell populations seen herein, we applied the TUNEL technique to examine spleen sections. Higher TUNEL-positive cell counts were observed in COVID-19 patients compared to CT (p= 0,04; **Figures 6A, B**).

Correlation analyses indicated that IL-6 and TNF^+^ WP cells, as well as TNF^+^ RP cells, were positively correlated with plasma cell accumulation in the RP and negatively correlated with T and B lymphocyte counts in the splenic WP (p ≤ 0.05; **Figure 6C**).

## 4 Discussion

The spleen plays a crucial role in immune response by either mounting an enduring immune response against microorganisms such as encapsulated bacteria, or by diverting the immune response, which favors infection (Mebius and Kraal, 2005). Both roles depend on morphological changes in the spleen compartments and the redistribution of cell populations (Silva et al., 2012; Silva-O’Hare et al., 2016; Hermida et al., 2018). Severe acute or chronic inflammation can provoke the disruption of splenic compartments, which may compromise the patient’s immune response and favor disease progression (Hermida et al., 2018). Here we show that severe COVID-19 led to the intense depletion of leukocyte populations in both RP and WP spleen compartments, in addition to marked reductions in the numbers and T and B lymphocytes; plasma cell counts increased in both compartments, while the number of macrophages was increased only in the WP. Together with cell population alterations, reticular fiber networks appeared fragmented, which further disrupts spleen structure and inhibits normal cell population homing. The densities of TNF- and IL-6-producing cells were increased in both splenic WP and RP.

The lymphocyte depletion observed in COVID-19 mirrors that seen in sepsis. Both conditions are characterized by a highly systemic inflammatory status with disproportionate leukocyte activation and death. Many mechanisms of cell death are activated in COVID-19. Apoptosis mediated by the FAS-FASL pathway, which is sustained by the continual stimulation of cytokines such as TNF and IL-6, may have been involved in the lymphocyte death seen in the spleens of the COVID-19 patients studied herein (Feng et al., 2020). The density of cells producing these two cytokines was increased, and the TUNEL analysis conducted revealed widespread cell death. Furthermore, elevated levels of these two cytokines have been negatively correlated with circulating lymphocyte counts and increased expression of T cell exhaustion markers in patients with COVID-19 (Diao et al., 2020; Arcanjo et al., 2021; Zhou et al., 2022). In sepsis, changes in the spleen play a crucial role in the unbalanced inflammatory response that results in immune system exhaustion and death (Cao et al., 2019). Furthermore, interventions such as splenectomy, immunomodulatory treatments, and the inhibition of apoptosis in the spleen have been shown to improve survival in experimental models of sepsis (Huston et al., 2008; Chen et al., 2021). It is important to note that all the COVID-19 patients in this study met the criteria for a diagnosis of sepsis. In fact, severe COVID-19 was reported as the leading cause of sepsis-related deaths from 2020 to 2022, surpassing bacterial sepsis (Karakike et al., 2021; Shappell et al., 2023). Of note, three of these patients also had evidence of coinfection by bacteria and fungus. Although consistent with other studies, we cannot rule out that some TUNEL-positive changes reflect the 5–8 h post-mortem interval, which represents a limitation of this study.

Viral infections alone can provoke disorganization in the spleen structure, as shown in patients with HIV infection or experimental models involving Rhesus Macaques or mice (Falk et al., 1988; Diaz et al., 2002; Moukambi et al., 2015; Samal et al., 2018). In the cases examined here, lymphoid compartments were scarcely visible, with indistinct structural organization. No secondary follicles were observed. In chronic infections such as visceral leishmaniasis, lymphoid follicle disorganization results in the impairment of lymphocyte migration into the lymphoid follicle secondary to follicular dendritic cell death, as well as decreased CXCL13 production (Silva et al., 2012). In such cases, evidence exists that lymphocyte repositioning is responsible for WP disorganization. In COVID-19, the absence of secondary lymphoid follicle formation may be due to the shift from traditional follicular helper differentiation to a Th1 phenotype in response to high expression of TNF in the tissue environment (Feng et al., 2020; Kaneko et al., 2020). Furthermore, the marked lymphocyte death seen in these fatal cases of COVID-19 was likely linked to lymphoid compartment atrophy and disorganization. The observed splenic disorganization and resulting disfunction may favor, together with the anergic state produced by extensive lymphoid cell death, secondary bacterial and fungus coinfections, which could have further aggravated the course of disease, ultimately resulting in death.

Despite extensive WP disorganization, pronounced plasmacytosis with the presence of some Mott cells was noted in the splenic RP. In chronic severe infections such as visceral leishmaniasis, WP disruption and late spleen plasmacytosis have been associated with extended plasma cell survival and splenic homing mediated by increased expression of *BAFF*, *APRIL* and *CXCL12* (Silva-O’Hare et al., 2016). It follows that changes in stromal and leukocyte cell populations in the spleen due to chronic inflammation may provoke alterations in gene expression patterns. It is possible that altered gene expression may not have occurred as a result of the short (14–26 days) disease duration in our patients with COVID-19. Therefore, the splenic plasmacytosis observed in these patients may be a remnant of a massive follicular antibody response or of continuous extrafollicular plasma cell differentiation. In fact, antibody responses against *SARS-CoV-2* infection can occur as early as one-week post-symptom onset, and IgM, IgG, and IgA antibody production has been documented at early stages of infection, possibly via the extrafollicular pathway (Woodruff et al., 2020; Lee et al., 2022). The lack of GC responses in COVID-19, due to increased TNF and IL-6 expression and the rapid progress of WP disruption, result in loss of hypermutation process, which may impair the production of high-affinity antibodies in patients with severe disease (Kaneko et al., 2020; Trevelin et al., 2022). Furthermore, increased levels of TNF and IL-6, key regulators of plasma cell survival, may contribute to the prolonged plasma cell persistence in the spleen (Khodadadi et al., 2019).

Macrophage density was increased in WP but not in the RP of patients with COVID-19. We cannot exclude the possibility that the increased density of these cells in the WP resulted from the extensively decreased lymphocyte population and derangement of the reticular framework of the atrophic lymphoid follicles. Nevertheless, the macrophages present in both spleen compartments appear to be less affected than the other cell types studied. Evidence suggests that the highly inflammatory environment in the spleen may be driven by infected macrophages and dendritic cells, and that these cells play a crucial role in the cytokine storm phenomenon, resulting in lymphopenia (Feng et al., 2020; Merad and Martin, 2020; Abdullaev et al., 2021; Xiang et al., 2021). Conversely, a hyper-responsive immune system in COVID-19 may lead to the excessive activation and proliferation of macrophages, known as macrophage activation syndrome (MAS) (McGonagle et al., 2021). This condition is associated with high levels of ferritin in the blood, blood clotting disorders, splenomegaly and hemophagocytosis (Chen et al., 2023). Three of our patients had extensive iron deposits in the spleen, and all three presented with microcirculatory thrombosis and erythrophagocytosis was evidenced in at least one patient.

Another aspect of spleen disorganization observed in COVID-19 is extensive reticular fiber fragmentation. The spleen is among the organs possessing high regenerative capability. During the regenerative process, proper cell positioning is dependent upon the extracellular matrix. Therefore, the disruption of the splenic framework may constitute an additional impairment to structural regeneration. In severe cases of COVID-19, patients with acute respiratory distress syndrome (ARDS) and low oxygen levels present high levels of metalloproteinases, which can break down the supportive tissue in the lungs, leading to serious organ damage (Zhu et al., 2022; Ma et al., 2023). Similarly, in nervous tissue, elevated cytokine levels, including IL-6—which correlates with cytokine levels observed in the spleen—have been associated with increased protease activity and tissue injury (Fekete et al., 2025). Similar changes may occur in the extracellular matrix of the spleen (Guizani et al., 2021; Lee and Kim, 2022). Alternatively, the cells responsible for producing the fibers in this framework may be directly affected by infection or changes in the splenic environment (Mueller and Germain, 2009; Morgado et al., 2020). Notably, a similar pattern of reticular fiber disruption has also been observed in the spleens of patients with sepsis, likely due to high metalloproteinase activity (Gunia et al., 2006).

In conclusion, the data presented here suggest that the spleen plays a significant role in the inflammatory state seen in COVID-19, whether through direct infection of the organ by *SARS-CoV-2* or via systemic inflammation. The observed changes in the spleen are consistent with the patients’ impaired capability to combat the primary infection by *SARS-CoV-2*, as well as coinfections.

## Data Availability

The raw data supporting the conclusions of this article will be made available by the authors, without undue reservation.
