# Supplementary material for: Lymphocyte loss and plasmacytosis are associated with IL-6- and TNF-producing cells in the spleens of fatal COVID-19 cases
Source: Front Cell Infect Microbiol. 2025 Oct 23;15:1645378. doi: 10.3389/fcimb.2025.1645378 (PMC12588937; doi:10.3389/fcimb.2025.1645378)
Supplement: Supplementary file 1 [file Table1.docx]

Supplementary Material

# Supplementary Table

**Supplementary Table 1.** Demographic and clinical data of control patients (CT, n=5) who underwent splenectomy at Hospital do Subúrbio, Salvador, Bahia-Brazil (2018-2021).

|  | **CT1** | **CT2** | **CT3** | **CT4** | **CT5** |
| --- | --- | --- | --- | --- | --- |
| **Sex** | M | F | M | M | M |
| **Age (years)** | 41 | 58 | 51 | 19 | 88 |
| **Spleen weight** | 245g | 76,5g | 59,4g | 60g | 82,25 |
| **Cause of death** | NA | Hemorrhagic shock | NA | Septic shock | NA |

NA= not applicable.
