# Supplementary figures and images for: Lymphocyte loss and plasmacytosis are associated with IL-6- and TNF-producing cells in the spleens of fatal COVID-19 cases
Source: Front Cell Infect Microbiol. 2025 Oct 23;15:1645378. doi: 10.3389/fcimb.2025.1645378 (PMC12588937; doi:10.3389/fcimb.2025.1645378)

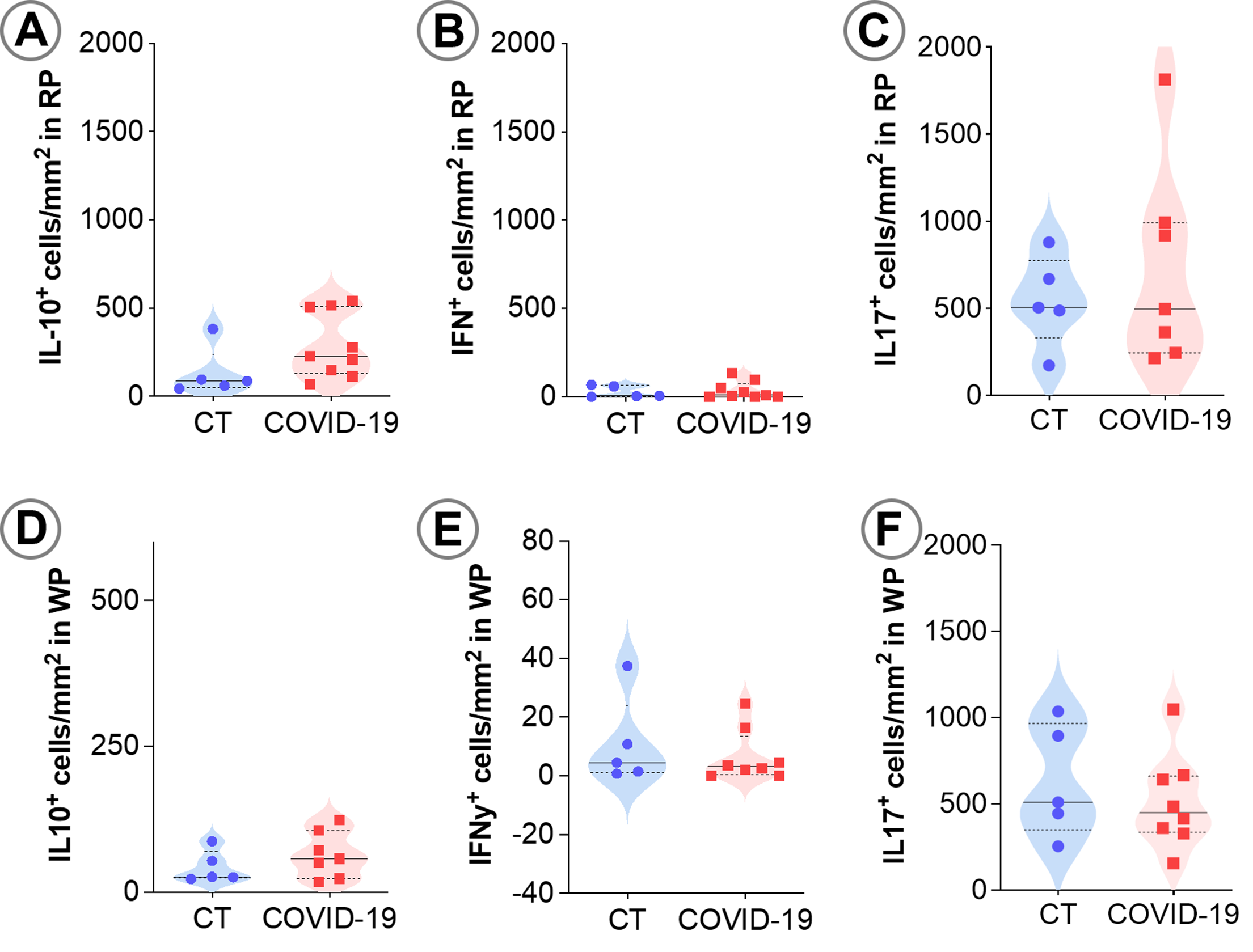

Supplement: Supplementary file 2 [file Image1.tif]
